# Elucidating the Influence of Gold Nanoparticles on the Binding of Salvianolic Acid B and Rosmarinic Acid to Bovine Serum Albumin

**DOI:** 10.1371/journal.pone.0118274

**Published:** 2015-04-10

**Authors:** Xin Peng, Wei Qi, Renliang Huang, Rongxin Su, Zhimin He

**Affiliations:** 1 School of Life Sciences, Tianjin University, Tianjin 300072, PR China; 2 Chemical Engineering Research Center, School of Chemical Engineering and Technology, Tianjin University, Tianjin 300072, PR China; 3 State Key Laboratory of Chemical Engineering, Tianjin University, Tianjin 300072, PR China; 4 Tianjin Key Laboratory of Membrane Science and Desalination Technology, Tianjin University, Tianjin 300072, PR China; 5 Collaborative Innovation Center of Chemical Science and Engineering (Tianjin), Tianjin 300072, P. R. China; 6 School of Environmental Science and Engineering, Tianjin University, Tianjin 300072, PR China; Aligarh Muslim University, INDIA

## Abstract

Salvianolic acid B and rosmarinic acid are two main water-soluble active ingredients from *Salvia miltiorrhiza* with important pharmacological activities and clinical applications. The interactions between salvianolic acid B (or rosmarinic acid) and bovine serum albumin (BSA) in the presence and absence of gold nanoparticles (Au NPs) with three different sizes were investigated by using biophysical methods for the first time. Experimental results proved that two components quenched the fluorescence of BSA mainly through a static mechanism irrespective of the absence or presence of Au NPs. The presence of Au NPs decreased the binding constants of salvianolic acid B with BSA from 27.82% to 10.08%, while Au NPs increased the affinities of rosmarinic acid for BSA from 0.4% to 14.32%. The conformational change of BSA in the presence of Au NPs (caused by a noncompetitive binding between Au NPs and drugs at different albumin sites) induced changeable affinity and binding distance between drugs and BSA compared with no Au NPs. The competitive experiments revealed that the site I (subdomain IIA) of BSA was the primary binding site for salvianolic acid B and rosmarinic acid. Additionally, two compounds may induce conformational and micro-environmental changes of BSA. The results would provide valuable binding information between salvianolic acid B (or rosmarinic acid) and BSA, and also indicated that the Au NPs could alter the interaction mechanism and binding capability of drugs to BSA, which might be beneficial to understanding the pharmacokinetics and biological activities of the two drugs.

## Introduction

In the past few years, there has been a rapid growth in the research of nanomaterials, which can be used in biotechnological, biomedical and industrial fields [1]. Nanoparticles are expected to form the basis of many of the technological and biological innovations of this century [2,3]. To date, a large number of nanoparticles have been synthesized, especially those made from noble metals such as gold. Gold nanoparticles (Au NPs) that possess novel optical, electronic and chemical properties have potential applications in chemistry and life sciences because of convenient surface bioconjugation with molecular probes and remarkable plasmon resonant optical properties [4]. Especially in modern biological and medical studies Au NPs have been widely employed, including genomics, biosensor, immunoassay, laser phototherapy of cancer cells and tumors, immune response enhancement, the targeted delivery of drugs and optical bioimaging et al [5,6]. Thus the influence of Au NPs targeted to human is inevitable as they can enter into the body through inhalation or ingestion from the lung or other organs. Furthermore, the small size Au NPs are similar to that of most biological molecules, and Au NPs can interact with many biological molecules [7,8]. Therefore, it is vital to understand the detailed reaction mechanism between Au NPs and biomolecules, especially the potential biological effect of Au NPs on the other life activities *in vivo*.

**Fig 1:**
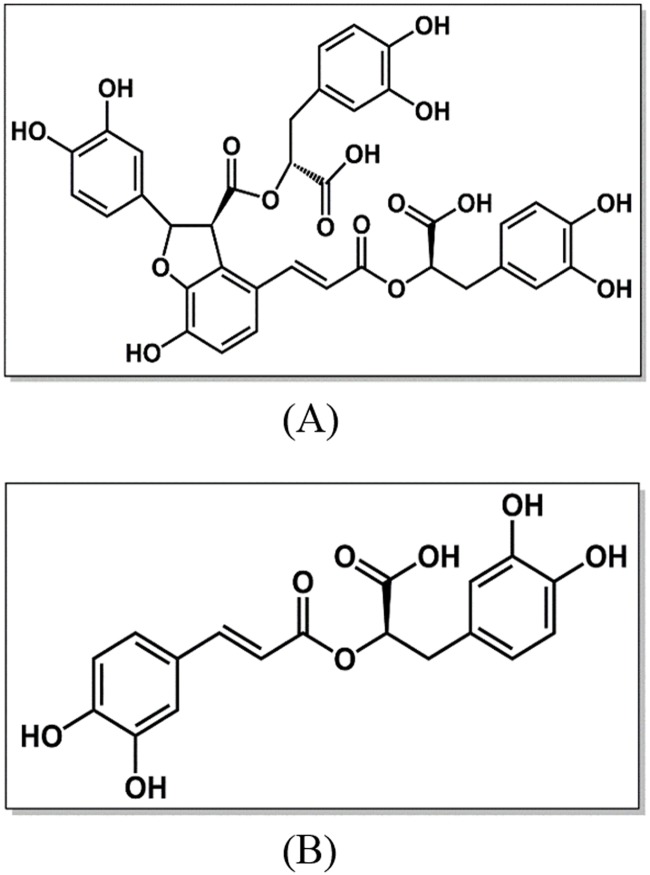
Molecular structures of salvianolic acid B (A) and rosmarinic acid (B).

*Salvia miltiorrhiza*, commonly named “Danshen” in China, is a well-known traditional Chinese medicinal herb and has been widely used to treat cardiovascular diseases for hundreds of years [9, 10]. Using chromatographic fingerprinting method and mass spectrometry, more than seventy compounds have been isolated and structurally identified from *S*. *miltiorrhiza* with various concentrations, which can be divided into water-soluble phenolic compounds and lipid-soluble diterpenoidal components [10]. The water-soluble compounds are mainly phenolic acid chemicals, including single phenolic acids and polyphenolic acids. Among polyphenolic acids, salvianolic acid A, salvianolic acid B, rosmarinic acid and lithospermic acid are the abundant components [11]. Here, we mainly focus on salvianolic acid B and rosmarinic acid. The molecular structures of the two antioxidant components are shown in Fig 1. Salvianolic acid B (SAB), the most abundant and bioactive compound in extract, has been shown to exert several pharmacological activities [12]. Studies have shown that SAB could inhibit Aβ peptide-induced neuronal toxicity [13], promote the angiogenesis and improve skin flap survival [14], attenuate brain damage and inflammation after traumatic brain injury [15], improve the recovery of motor function after cerebral ischemia [16], and selectively inhibit the activities of matrix metalloproteinase-9 and efficiently prevent cardiac remodeling [17]. Rosmarinic acid (RA), a well-known hydroxycinnamic acid ester, is an importantly natural occurring phenolic bioactive compound and can be used as a food additive [18]. In addition, it also possesses a number of significantly biological activities [18]. Previous studies demonstrated that RA could protect the keratinocytes [19], reduce the atopic dermatitis [20], prevent the Alzheimer’s disease [21] and inhibit the activities of HIV-1integrase [22].

Serum albumin (SA) is a kind of principally soluble protein in the circulatory system with a concentration of 0.6–0.7 mM and has many physiological functions including as transporters for a variety of organic and inorganic compounds and maintaining the osmotic pressure and pH of blood [23]. Bovine serum albumin (BSA) is composed of 583 amino acid residues [24]. Analysis of crystal structure has revealed that BSA is composed of a singly large α–helix polypeptide chain with 17 disulfide bridges, and contains three structural domains (I–III) and numerous potential drug binding sites. BSA contains two tryptophan residues, Trp-134 and Trp-212, of which the former is located in hydrophilic subdomain IB, and the latter in hydrophobic subdomain IIA [25]. BSA has been one of the most extensively studied proteins, not only because of its medical importance, low cost, availability, ligand-binding properties and wide acceptance in the pharmaceutical industry, but also due to its structural homology with human serum albumin [26]. In pharmaceutical industry it is important to determine and characterize the pharmacokinetics and pharmacological effects of drugs. It is universally considered that the effectiveness of drugs depends on their binding ability to proteins. The binding affinity also affects the rate at which the drug is released to sites of catabolism or pharmacological action. Thus, analysis of the drug binding to serum albumin is interesting not only because the unbound drug fraction affects many critical pharmacokinetic parameters including the steady-state distribution volume, but also because it provides important information to design the administration regimen dose [27–29]. Hence, it is important to note that serum albumin can ultimately affect the drug’s ADME (absorption, distribution, metabolism and excretion) properties. SA has a number of binding sites for various endogenous and exogenous ligands and is also a flexible molecule, thus it can simultaneously bind several ligands [30]. Generally, the use of a combination of several drugs is often necessary, especially during long-term therapy. But using several compounds simultaneously can cause a change in the amount of a particular drug bound to plasma proteins. That is to say, drug binding to plasma proteins can be altered by the concurrent administration of other drugs with similar physicochemical characteristics that compete with each other and with endogenous substances for common or functionally linked binding sites. Likewise, the use of Au NPs, which can enter the body through the lungs or other organs *via* medicine, food and drink and are able to adsorb the SA, could modify the interaction between SA and drug. The binding of a drug to SA can be weakened or enhanced in the presence of other drugs or Au NPs as a result of an overlap of binding sites and/or conformational changes. Hence, competitive and allosteric mechanisms can modulate drug binding to plasma proteins. It is therefore necessary to employ a monitoring therapy owing to the possible increase of uncontrolled toxic effects. In addition, a detailed characterization of serum albumin binding properties is also necessary not only for understanding its key physiological functions, but also most recently to facilitate development as a designed delivery vehicle for a range of therapeutic and diagnostic drugs [30]. Therefore, the topic about drug-protein interaction has become the hot and central issue in the fields of medicine, chemistry and life science [31].

Recently, the Au NPs-proteins and small compounds-proteins interactions have attracted great interests, but how Au NPs affect the interaction between drugs and BSA is not clear. Herein, the influence of Au NPs with different diameters on the affinity of SAB/RA to BSA was investigated. Some characteristic data of the interaction, such as binding constant, the stoichiometry of binding, the binding distance and the conformational change of BSA were obtained. Moreover, a comparative study between BSA-SAB and BSA-RA was performed in order to understand the interaction mechanism of protein-drug complex.

## Materials and Methods

### Materials

BSA (≥98%) and ibuprofen were purchased from Sigma–Aldrich Chemical Company (Shanghai, China) and used without further purification. Salvianolic acid B (≥98%, analytical standard, SAB), rosmarinic acid (≥97%, analytical standard, RA), tetrachloroauric acid (HAuCl_4_ 3H_2_O), sodium citrate, warfarin and digitoxin were purchased from Aladdin Reagent Co., Ltd. (Shanghai, China). The stock solution of BSA (1.0 × 10^-5^ mol L^-1^) was directly dissolved in 0.02 mol L^-1^ phosphate buffer of pH 7.4 with 0.1 mol L^-1^ NaCl and was kept in a refrigerator at 4°C. The pH measurements were carried out with a digital pH-meter with a combined glass-calomel electrode. The average molecular weight value of 66000 g mol^-1^ was used in the preparation of BSA solutions. The stock solutions of SAB and RA drugs (1.0 × 10^-4^ mol L^-1^) were prepared by dissolving relevant crystals with phosphate buffer solution (PBS), and PBS was used as blank for all the samples in this study. All solutions were degassed in an ultrasonic bath prior to use. All the other chemicals were of analytical reagent grade and double–distilled water was used throughout all the experiments.

### Preparation and Characterization of Au NPs

Prior to the preparation of Au NPs, all glass wares used in the experiment were cleaned in a bath of freshly prepared aqua regia solution (HCl/HNO_3_, 3:1), rinsed thoroughly with double-distilled water, and then oven-dried before use. The citrate-stabilized Au NPs were synthesized based on the classical Frens-Turkevich method [32]. In a typical experiment, a 1.25 mL sample of aqueous HAuCl_4_ (1.0 × 10^-2^ mol L^-1^) and corresponding double-distilled water were added to a 100 mL flask. The solution was brought to boil while being stirred; afterward the corresponding amount of aqueous sodium citrate (1.0 × 10^-1^ mol L^-1^) with certain initial molar ratio of citrate to Au^3+^ was added rapidly. The total reaction volume was 50 mL. The vigorously stirred mixture was allowed to heat under reflux for 10 min, and the color of the solution was changed from pale yellow to wine red. To ensure complete reduction, stirring was continued for another 30 min without heating, and was slowly cooled down to room temperature. The reactions in the series of variable Na_3_Ct/HAuCl_4_ ratios were performed by varying the concentration of Na_3_Ct with a fixed HAuCl_4_ concentration. All solutions were clarified by ultrafiltration through 0.2 μm filters (Millipore Corp., USA). The resulting Au NPs were stored at 4°C in the dark to minimize photo induced oxidation.

UV-vis absorption spectra for Au NPs were recorded with a TU–1810SPC spectrophotometer (Puxi General Instrument Ltd. of Beijing, China) in the wavelength range from 300 to 800 nm, with a resolution of 1 nm. The TEM images of Au NPs were taken on a JEOL-TEM-2010 transmission electron microscope (JEOL Ltd, Japan) with an operating voltage of 200 kV. TEM samples were prepared by dropping Au NPs solution onto a copper grid covered with Formvar film. The size distribution of Au NPs was obtained by averaging the sizes of at least 300 particles directly from the TEM images.

### UV–vis Absorption Spectroscopy Measurements

The UV–vis absorption spectra were obtained on a TU–1810SPC spectrophotometer (Puxi General Instrument Ltd. of Beijing, China) equipped with a 1.0 cm path length cell. The samples were incubated for 30 min and the spectra were measured in the range of 200–500 nm at room temperature. The UV-vis absorption spectra of drugs (5.0 × 10^-6^ mol L^-1^ and 25.0 × 10^-6^ mol L^-1^) and the drug–Au NPs systems (drug, 25.0 × 10^-6^ mol L^-1^ and Au NPs, 10.0 × 10^-11^ mol L^-1^) were measured, respectively.

### Fluorescence Spectroscopy Measurements

All fluorescence spectra were performed on a Cary Eclipse Spectrofluorimeter (Varian corporate, USA) equipped with a 1.0 cm path-length quartz cell. A circulating water bath was used to keep the temperature needed. The widths of both of the excitation slit and the emission slit were set at 5.0 nm for all the measurements. 280 nm has been chosen as excitation wavelength, and the emission fluorescence were obtained from 290 to 500 nm at 298 K. The 3.0 mL solutions of 5.0 × 10^-6^ mol L^-1^ BSA with or without Au NPs (10.0 × 10^-11^ mol L^-1^) were incubated with various volume of drugs to make the concentration ratio of drug and protein to be 0, 0.5, 1, 1.5, 2, 2.5, 3, 4 and 5, respectively. Titrations were done manually by using trace syringe. All solutions were mixed thoroughly and kept 10 min before measurements. It is important to note that, in the course of increasing concentrations of the drugs, an instrumental inner filter effect (IFE) would cause some decrease in the fluorescence emission intensity. This effect is an inherent problem of many fluorimetric procedures which can lead to the results departed from the initial linearity and therefore must be taken into account. To avoid inner filter effect caused by adsorption of exciting light and reabsorption of the emitted light, we calculated the sum of absorbance at excitation wavelength (280 nm) and the emission wavelength (about 349 nm) under different conditions to eliminate the effect. Hence, the fluorescence intensities can be corrected in the light of the following equation (Eq (1)) [33]:

$$F_{cor}=F_{obs}e^{\left(A_{ex}+A_{em}\right)/2}$$ (1)

where *F*_*cor*_ and *F*_*obs*_ are the fluorescence intensities corrected and observed, respectively; and *A*_*ex*_ and *A*_*em*_ represent the absorption of the system at the excitation and emission wavelengths, respectively. The fluorescence intensities utilized in this study were the corrected data.

The competitive experiments were performed at 298 K using three different site markers, warfarin for site I, ibuprofen for site II, and digitoxin for site III. Three milliliters of BSA-drug mixture solution (BSA: 5.0 × 10^-6^ mol L^-1^, SAB/RA: 2.0 × 10^-5^ mol L^-1^) was added to a quartz cell. Then the site markers stock solutions were successively added to the mixture solution to obtain an overall site marker concentration ranging from 5 to 40.0 × 10^-6^ mol L^-1^. The fluorescence spectra were recorded in the range of 290–500 nm when the excitation wavelength was 280 nm. Fluorescence was then calculated as percentage of the initial fluorescence.

The synchronous fluorescence spectroscopy was obtained to investigate the conformational changes of protein at 298 K, while the wavelength intervals between excitation and emission wavelengths (Δ*λ*) were fixed at 15 and 60 nm, and the fluorescence spectra were recorded in the wavelength range of 250–330 nm and 245–330 nm, respectively.

Three-dimensional fluorescence measurements of BSA (5.0 × 10^-6^ mol L^-1^) and the drug-BSA system (molar ratio of 2:1) with or without Au NPs (10.0 × 10^-11^ mol L^-1^) were measured at the following conditions: the emission spectra were recorded from 200 nm to 360 nm and the number of scanning curves was 17; the excitation wavelengths were in the range from 230 nm to 550 nm with 10nm interval, and the other scanning parameters were just the same as those for the fluorescence spectra of BSA.

### Statistical Analysis

The least-squares method was used to fit functions through a regression analysis. Statistical analyses were performed with GraphPad Prism (version 5.00, GraphPad Software, San Diego, CA, USA). Analyses of variance (ANOVA) and Tukey’s HSD test with a significance level of 0.05 were applied.

## Results and Discussion

### Characterization of Au NPs

**Fig 2:**
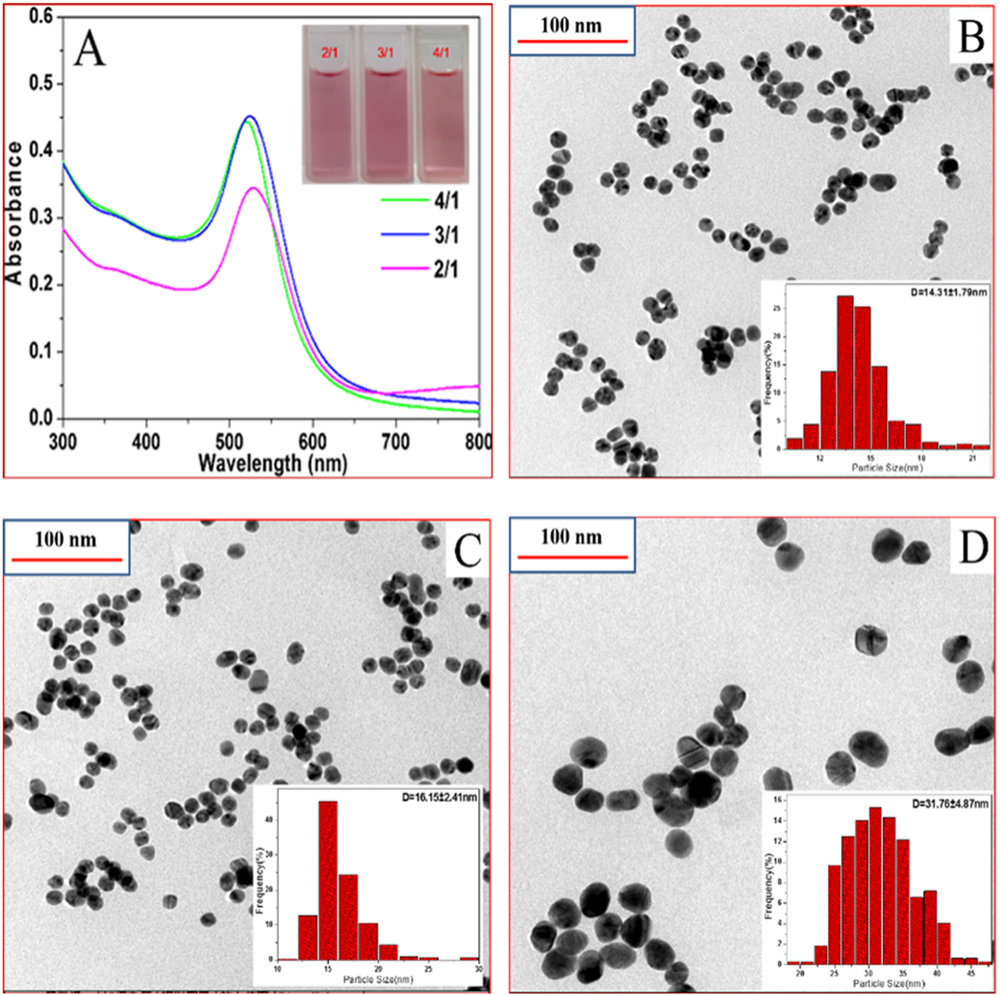
Characterization of Au NPs. (A) UV-visible absorbance spectra of Au NPs. *Inset* is the photo taken from gold hydrosols, (B-D) Representative TEM images and the corresponding size distributions (*inset*) of Au NPs synthesized at different molar ratio of sodium citrate/chloroauric acid. (B) 4/1, (C) 3/1, (D) 2/1.

The synthesized Au NPs solutions exhibited a color of reddish brown (inset in Fig 2A), which was known to arise from the collective oscillation of the free conduction electrons induced by an interacting electromagnetic field. The UV-vis absorption spectra of Au NPs solutions (Au4, Au3, Au2) at different molar ratio of sodium citrate to chloroauric acid (4/1, 3/1, 2/1) were shown in Fig 2A. It could be found that the maximum wavelength of the surface plasmon resonance (SPR) displayed an obvious red-shift with the gradually decreasing molar ratio (from 4/1 to 2/1). The maximum absorption peaks of Au4, Au3 and Au2 were 519.5 nm, 524.0 nm and 528.5 nm, respectively. Because the maximum absorption peak bore a linear relationship with the size of the nanoparticles, the Au2 had the maximum particle diameter. In order to investigate the size and the shape of Au NPs and to confirm their aggregation, we used TEM to measure the nanoparticles. Fig 2B–2D depicted the TEM images of different-sized Au NPs and the particle size histograms. It could be seen that the prepared Au NPs were almost spherical shaped and separated from each other. Furthermore, Au NPs had a narrow size distribution, and the average diameters of Au4, Au3 and Au2 were 14.31 nm, 16.15 nm and 31.76 nm, respectively, as estimated by analyzing the images represented the overall size distribution (inset in Fig 2B–2D).

### Fluorescence Quenching Spectroscopy

Fluorescence is the photon emission process caused by the return of an electron from a higher energy orbital to a lower orbital. Many kinds of molecular interactions could induce fluorescence quenching, such as excited–state reaction, molecular rearrangement, energy transfer, ground state complex formation and collisional quenching [34]. Fluorescence spectroscopy is widely used in biochemical, medical, material and chemical research fields. For proteins, the fluorescence measurements can provide some understanding of the binding of small molecule ligands to protein at the molecular level. This is because almost all proteins contain naturally fluorescent amino acid residues such as tyrosine and tryptophan. When the proteins bind to other substances or the conformation of protein is changed, the corresponding fluorescence spectroscopy intensity and/or the wavelength of the maximum peak of the protein will generate some alterations [35,36]. Fluorescence spectroscopy is one of the traditional methods to investigate the properties of proteins such as stability, hydrodynamics, kinetics and ligand binding because of its exquisite sensitivity and relative ease of use [30,37].

In order to identify the participation of tyrosine and tryptophan groups in the binding process between SAB/RA and BSA in the absence and presence of Au NPs, the fluorescence of BSA excited at different wavelengths were determined in the presence of SAB/RA. The previous research suggested that the tyrosine and tryptophan residues in serum albumin were excited at 280 nm wavelength, while at an excitation wavelength of 295 nm, only the tryptophan residue emitted fluorescence [38]. The plots of *F*/*F*_o_ against [drug]/[BSA] were presented in Figure A in S1 File, where *F*_o_ and *F* were the fluorescence intensities before and after the addition of SAB/RA, respectively. It can be found that no significant difference was observed in the quenching of BSA fluorescence between two excitation wavelengths 280 and 295 nm. This indicated that only the tryptophan residue was implicated in the fluorescence quenching and that tyrosine residue did not participate in the molecular interaction between SAB/RA and BSA without and with Au NPs. It also supported the idea of domain II being one of the possible interaction sites of SAB/RA with BSA [39,40].

**Fig 3:**
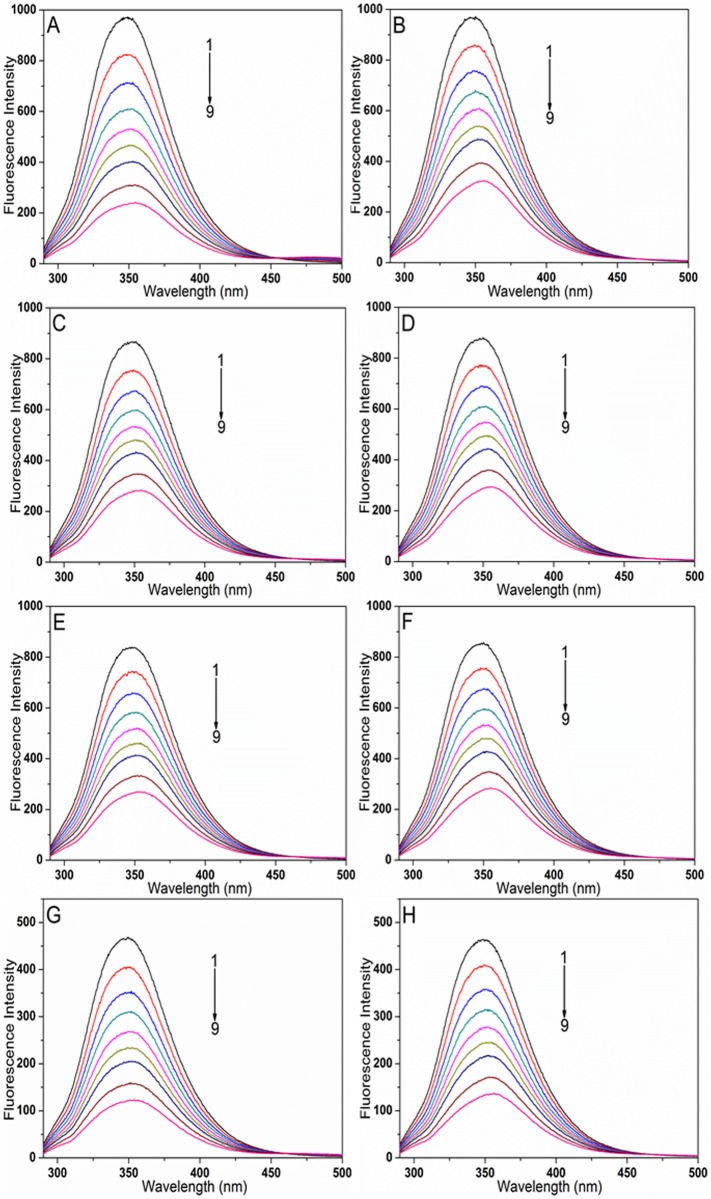
Fluorescence quenching spectra of BSA–drug systems with and without Au NPs at 298 K, *λ*_ex_ = 280 nm, C_BSA_ = 5.0 × 10^-6^ mol L^-1^. (A) BSA-SAB system, the concentrations of SAB (1–9) were 0, 2.5, 5.0, 7.5, 10.0, 12.5, 15.0, 20.0, and 25.0 × 10^-6^ mol L^-1^, respectively. (B) BSA-RA system, the concentrations of RA (1–9) were 0, 2.5, 5.0, 7.5, 10.0, 12.5, 15.0, 20.0, and 25.0 × 10^-6^ mol L^-1^, respectively. (C) BSA-SAB-Au4 system, C_Au4_ = 10.0 × 10^-11^ mol L^-1^, the concentrations of SAB (1–9) were 0, 2.5, 5.0, 7.5, 10.0, 12.5, 15.0, 20.0, and 25.0 × 10^-6^ mol L^-1^, respectively. (D) BSA-RA-Au4 system, C_Au4_ = 10.0 × 10^-11^ mol L^-1^, the concentrations of RA (1–9) were 0, 2.5, 5.0, 7.5, 10.0, 12.5, 15.0, 20.0, and 25.0 × 10^-6^ mol L^-1^, respectively. (E) BSA-SAB-Au3 system, C_Au3_ = 10.0 × 10^-11^ mol L^-1^, the concentrations of SAB (1–9) were 0, 2.5, 5.0, 7.5, 10.0, 12.5, 15.0, 20.0, and 25.0 × 10^-6^ mol L^-1^, respectively. (F) BSA-RA-Au3 system, C_Au3_ = 10.0 × 10^-11^ mol L^-1^, the concentrations of RA (1–9) were 0, 2.5, 5.0, 7.5, 10.0, 12.5, 15.0, 20.0, and 25.0 × 10^-6^ mol L^-1^, respectively. (G) BSA-SAB-Au2 system, C_Au2_ = 10.0 × 10^-11^ mol L^-1^, the concentrations of SAB (1–9) were 0, 2.5, 5.0, 7.5, 10.0, 12.5, 15.0, 20.0, and 25.0 × 10^-6^ mol L^-1^, respectively. (H) BSA-RA-Au2 system, C_Au2_ = 10.0 × 10^-11^ mol L^-1^, the concentrations of RA (1–9) were 0, 2.5, 5.0, 7.5, 10.0, 12.5, 15.0, 20.0, and 25.0 × 10^-6^ mol L^-1^, respectively.

To gain an insight into the binding mechanism of SAB/RA to BSA affected by Au NPs, the fluorescence spectra of BSA with SAB/RA in the absence and presence of Au NPs of different sizes were investigated with an excitation wavelength of 280 nm (Fig 3). It was shown that BSA exhibited a strong fluorescence emission band at 349.07 nm at pH 7.4 and 298 K. As can be seen, its intensity decreased gradually with the addition of SAB/RA with or without Au NPs. Furthermore, the peak position and shape of the emission spectra with Au NPs at different concentrations of drug were similar to those without Au NPs, while the fluorescence quenching extent was larger than those without Au NPs. In addition, the drugs resulted in an obvious red-shift (about 3–8 nm) of the maximum emission of BSA whether there existed Au NPs or not, which indicated the microenvironment of the chromophore of BSA was changed [41]. All of these spectral changes suggested that SAB/RA could interact with BSA and quench its intrinsic fluorescence in both the presence and absence of three different sizes of Au NPs. In other words, the formation of a new complex between BSA and drug was observed. So as to better inspect the effect of Au NPs on the interaction, we calculated the fluorescence quenching extent of all systems when the concentration of drug was the maximum (25.0 × 10^-6^ mol L^-1^). Approximately 75.23% and 66.69% of fluorescence quenching were observed when the concentration of SAB/RA reached 25.0 × 10^-6^ mol L^-1^ in the absence of Au NPs. The results demonstrated that the quenching effect of drug on BSA fluorescence in the absence of Au NPs depended on their structures [42]. When three different sizes of Au NPs (Au4, Au3 and Au2) were added to two different systems (BSA-SAB or BSA-RA), the fluorescence decreased by 67.42% for BSA-SAB-Au4, 67.74% for BSA-SAB-Au3, 73.67% for BSA-SAB-Au2; 66.73% for BSA-RA-Au4, 66.77% for BSA-RA-Au3, 70.38% for BSA-RA-Au2. Thus, for the BSA-SAB system, the fluorescence quenching extent decreased with gradually decreasing size of the Au NPs; but for the BSA-RA system, the influence of Au NPs on the quenching extent of RA to BSA was determined as: Au4 < Au3 < Au2.

### The Mechanism of Fluorescence Quenching

**Fig 4:**
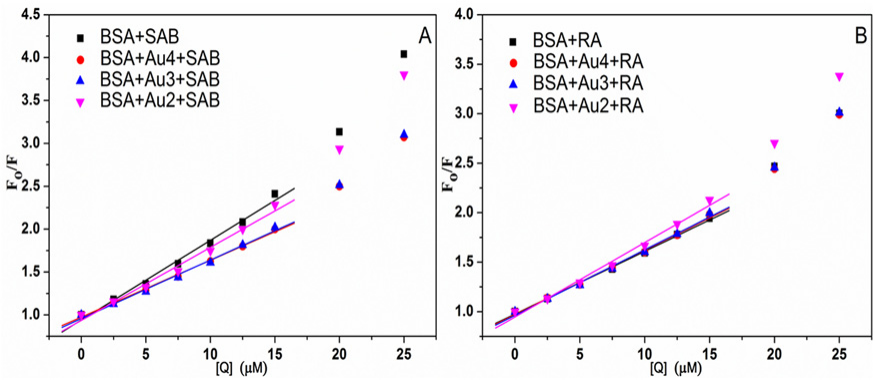
The Stern-Volmer plots of BSA–drug systems without and with Au NPs at 298 K. The Stern-Volmer plots for BSA fluorescence quenching by SAB (A) and RA (B) in the absence of Au NPs (square) and in the presence of Au4 (circle), Au3 (up triangle) and Au2 (down triangle). C_BSA_ = 5.0 × 10^-6^ mol L^-1^, the concentration of Au NPs was 10.0 × 10^-11^ mol L^-1^, *λ*_ex_ = 280 nm, pH = 7.40.

There are mainly two different types of fluorescence quenching mechanisms: dynamic and static quenching. In static quenching, it is caused by the formation of ground-state complex between the fluorophore and the quencher, and this complex is nonfluorescent; while in dynamic quenching, it is resulted from the collisional encounters between the excited-state fluorophore and the quencher [43]. To clarify the quenching mechanism between SAB/RA and BSA in the presence of Au NPs, we used the Stern-Volmer equation (Eq (2)) to analyze the fluorescence quenching data [34].

$$\frac{F_{o}}{F}=1+K_{SV}\left[Q\right]=1+k_{q}\tau_{o}\left[Q\right]$$ (2)

where *F*_o_ denotes the steady–state fluorescence intensity of BSA; *F* is the steady–state fluorescence intensity of BSA in the presence of drug with various concentrations; *K*_SV_ is the Stern–Volmer quenching constant for BSA; [*Q*] is the concentration of quencher; *k*_q_ is the quenching rate constant of BSA; *τ*_*o*_ is the average fluorescence lifetime of molecule without quencher and is generally equal to 5.78 ×10^-9^ s for biomacromolecules [44]. The plots of the Stern-Volmer equation at 298 K were displayed in Fig 4 and estimated parameters of Eq (2) were presented in Table 1. As shown in Fig 4, the Stern-Volmer plots largely deviated from linearity toward the y-axis at high concentrations of SAB and RA in all systems, which indicated that both dynamic and static quenching were involved for SAB/RA on BSA fluorescence [42]. In many cases this positive deviation from the Stern-Volmer equation showed that the fluorophore was quenched by a combined quenching (static and dynamic) procedure with the same quenchers. Nevertheless, in other cases, the upward curvature indicated that the presence of a sphere of volume around a fluorophore within which a quencher would cause quenching with a probability of unity [36]. In this situation, quenching occurred because of the quencher being adjacent to the fluorophore at the moment of excitation. These closely spaced fluorophore-quencher pairs were immediately quenched, but fluorophore and quencher did not actually form a ground-state complex. This type of apparent static quenching was usually interpreted in terms of the model “sphere of action” [36]. In this paper, to provide a semiempirical measure of the magnitude of the quenching in all research systems, the quenching in terms of *K*_SV_ and *k*_q_ values at low quencher concentrations with the nearly linear plots was investigated (Fig 4). The good fitting linearity with R^2^ > 0.99 (Table 1) suggested that the Stern-Volmer model was appropriate for studying the binding mechanism between SAB/RA and BSA. From the analysis of Table 1, all the values of *k*_q_ from the protein quenching procedures were much larger than the maximum scatter collision quenching constant of various kinds of quenchers to biopolymer (2.0 × 10^10^ mol L^-1^ s^-1^). So it meant that the quenching process was predominantly static [45], whereas the dynamic terms contributed significantly to quenching only at higher concentrations [33,43]. Consequently, a conclusion could be safely drawn that the dynamic quenching was not the dominant quenching mechanism at least at low SAB/RA concentrations, and that the interactions of the studied compounds with BSA in the presence and absence of Au NPs were characteristic of the combined quenching mechanism. Moreover, in the BSA-SAB system the addition of Au NPs would induce the decrease of quenching constants, in other words, Au NPs decreased the quenching constant from 28.67% to 8.47%, and the influence of Au NPs on the quenching constants of SAB for BSA was determined as: Au4 > Au3 > Au2; while in the BSA-RA system the presence of Au NPs improved the quenching efficacy of RA with BSA, in other words, Au NPs increased the quenching constant from 2.67% to 18.71%, and the quenching constants increased in order of Au4 < Au3 < Au2. These results suggested that the Au NPs had different behaviors for the dissimilar interaction systems, and both the drug structures and the size of Au NPs affected the binding interaction between drug and BSA [42].

**Table 1:** Stern-Volmer quenching constants (*K*_SV_) and bimolecular quenching rate constants (*k*_q_) of drug-protein systems with and without Au NPs at 298 K and pH = 7.4.

Compound	SAB			RA		
	*K*_SV_ (×10^4^ L mol^-1^) ^a^	*k*_q_ (×10^13^ L mol^-1^ s^-1^)	*R* ^2*b*^	*K*_SV_ (×10^4^ L mol^-1^) ^a^	*k*_q_ (×10^13^ L mol^-1^ s^-1^)	*R* ^2*b*^
BSA	9.30 ± 0.05	1.61 ± 0.29	0.9951	6.33 ± 0.02	1.10 ± 0.12	0.9987
BSA-Au4	6.63 ± 0.02	1.15 ± 0.12	0.9982	6.50 ± 0.03	1.13 ± 0.17	0.9977
BSA-Au3	6.83 ± 0.04	1.18 ± 0.21	0.9963	6.62 ± 0.03	1.15 ± 0.17	0.9969
BSA-Au2	8.51 ± 0.06	1.47 ± 0.34	0.9935	7.52 ± 0.04	1.30 ± 0.22	0.9953

^a^ Mean values are statistically different (P < 0.05). (ANOVA and Tukey’s HSD test).

^b^ R^2^ is the correlation coefficient.

### Analysis of the Binding Constants and the Stoichiometry of Binding

**Fig 5:**
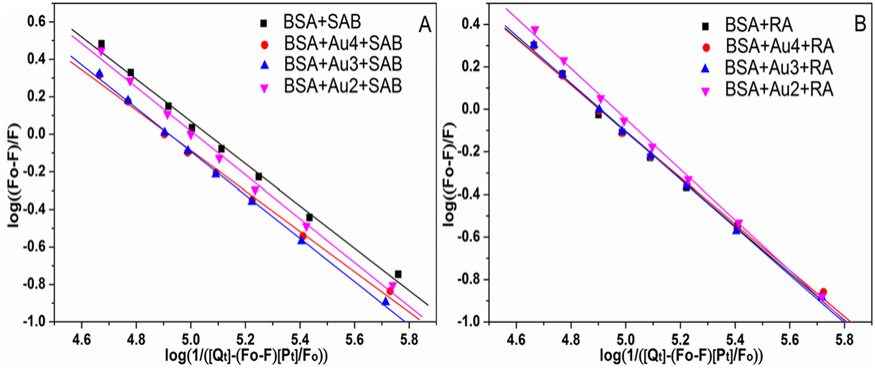
Plots for calculating the binding constants and the stoichiometry of binding of BSA–drug systems without and with Au NPs. Double logarithmic curves of SAB (A) and RA (B) quenching BSA fluorescence in the absence of Au NPs (square) and in the presence of Au4 (circle), Au3 (up triangle) and Au2 (down triangle). C_BSA_ = 5.0 × 10^-6^ mol L^-1^, the concentration of Au NPs was 10.0 × 10^-11^ mol L^-1^, *λ*_ex_ = 280 nm, pH = 7.40.

The value of binding constant (*K*_b_) that describes the binding ability of ligand to protein is helpful to understand the bio-distribution state of drug within plasma. The binding degree of drug-plasma protein can give significant information on the pharmacokinetic and the pharmacodynamics properties of a drug or better it can determine and specify the drug distribution. High degree of the protein binding can prolong the drug duration of action, decrease the concentration of free drug and compete with other agents for the same protein binding site, and vice versa [46,47]. The interactions between SAB/RA and BSA may be affected by Au NPs in the body. The effects of Au NPs on binding constants of BSA-drug complex were investigated at 298 K. In order to analyze thoroughly the equilibrium between free and bound molecules and rationalize our experimental data on the BSA-drug systems, the following equation (Eq (3)) has been employed [48]:

$$\text{log}\left(F\text{o}-F\right)/F=n\text{log}K_{b}-n\text{log}\left(1/\left(\left[Q_{t}]-\left(Fo-F\right)[P_{t}\right]/Fo\right)\right)$$ (3)

where *F*_o_ and *F* are the fluorescence intensities before and after the addition of the quencher; *K*_b_ is the binding constant; *n* is the stoichiometry of binding [44]; [*Q*_t_] and [*P*_t_] are the total ligand concentration and total protein concentration, respectively. According to Eq (3), *K*_b_ and *n* values can be obtained by the plot of log (*F*_o_-*F*)/*F* versus log(1/([*Q*_t_]-(*F*_o_-*F*)[*P*_t_]/*F*_o_)) (Fig 5). The values of *n* and *K*_b_ at 298 K were listed in Table 2. For all systems, the binding constants (*K*_b_) of BSA-drug were more or less at 10^5^ order, and the stoichiometry of binding (*n*) in BSA approximated to 1, indicating that there existed higher affinity of drugs for BSA and only one site in BSA was reactive to BSA-drug complex [48]. It could be seen that Au4, Au3, Au2 obviously reduced the affinity of SAB for BSA about 27.81%, 27.63% and 10.08%, respectively, which meant that the decreasing percentage of *K*_b_ value for BSA increased with the decreasing size of Au NPs. On the contrary, the *K*_b_ value for BSA-RA system increased when Au NPs were added and the increasing extent depended on the size of Au NPs (Au4 of 0.40%, Au3 of 1.05%, Au2 of 14.32%). Thus, these results led to the conclusion that the interaction behavior between drugs and BSA was changed after the addition of Au NPs to the BSA-drug complex. As shown in Table 2, the affinity of SAB to BSA was greater than that of RA in all four systems. Compared with RA, the molecular structure of SAB possessed more hydroxyl groups, so the SAB may form more hydrogen bond with the hydroxyl groups in BSA, which improved the binding affinity between SAB and BSA.

**Table 2:** Binding constants (*K*_b_), the stoichiometry of binding (*n*) and free energy change (Δ*G*) of drug-protein systems with and without Au NPs at 298 K and pH = 7.4.

Compound	SAB			RA		
	*K*_b_ (×10^4^ L mol^-1^) ^a^	*n*	Δ*G* (kJ mol^-1^)	*K*_b_ (×10^4^ L mol^-1^) ^a^	*n*	Δ*G* (kJ mol^-1^)
BSA	11.52 ± 0.03	1.13 ± 0.02	-28.89 ± 0.14	8.02 ± 0.03 ^c^	1.11 ± 0.02	-27.99 ± 0.14
BSA-Au4	8.31 ± 0.03 ^b^	1.08 ± 0.02	-28.08 ± 0.13	8.05 ± 0.03 ^c^ ^d^	1.09 ± 0.02	-28.00 ± 0.12
BSA-Au3	8.33 ± 0.02 ^b^	1.16 ± 0.01	-28.08 ± 0.11	8.10 ± 0.02 ^d^	1.13 ± 0.01	-28.02 ± 0.13
BSA-Au2	10.35 ± 0.03	1.17 ± 0.02	-28.62 ± 0.14	9.17 ± 0.02	1.19 ± 0.03	-28.32 ± 0.11

^a^ Mean values are statistically different (P < 0.05). (ANOVA and Tukey’s HSD test).

^b^ Mean values are statistically similar (P > 0.05).

^c^ Mean values are statistically similar (P > 0.05).

^d^ Mean values are statistically similar (P > 0.05).

Free energy change ΔG can be estimated from the following equation (Eq (4)) based on the binding constant K_b_ [26]:

$$\Delta G=-RT\text{ln}K_{b}$$ (4)

where *T* is the absolute temperature and *R* is the universal gas constant. Δ*G* reflects the possibly of reaction. The negative sign for Δ*G* meant the binding process of all systems was spontaneous, independent of the addition of Au NPs [26].

### Energy Transfer from BSA to SAB and RA

Plenty of information concerning the molecular details of donor-acceptor pair can be gained from non-radiative energy transfer. Fluorescence resonance energy transfer (FRET) is a non-radiative transfer of the excitation energy from a donor to an acceptor chromophore. It is mediated by a long-range interaction between the emission and absorption transition dipole moments of the donor and the acceptor, respectively. FRET is also a good technique to evaluate the distance (in nanometer scale) between the donor (fluorophore) and acceptor *in vitro* and *in vivo* [49,50]. According to Förster’s non–radiation energy transfer theory, energy transfer will happen at the following conditions: (a) the donor can produce fluorescence; (b) the donor’s fluorescence emission spectrum and the acceptor’s UV–vis absorbance spectrum have more overlap; (c) the distance between the donor and the acceptor is no more than 8 nm [51].

The distance *r* between SAB/RA and BSA can be calculated by the following Eq (5):

$$E=1-\frac{F_{o}}{F}=\frac{R_{o}^{6}}{R_{o}^{6}+r^{6}}$$ (5)

where *E* represents the energy transfer efficiency between the donor (BSA) and the acceptor (SAB/RA); *R*_o_ is the critical distance when the energy transfer efficiency is 50% and it can be calculated from donor emission and acceptor absorption spectra by using the Förster’s formula (Eq (6)):

$$R_{o}^{6}=8.79\times 10^{-25}K^{2}N^{-4}ФJ$$ (6)

where *K* ^2^ is the spatial orientation factor of the dipole and equals to 2/3 for random orientation as in fluid solution; *N* is the average refractive index of medium; *Φ* is the fluorescence quantum yield of the donor; *J* is the integral of the spectral overlap between the emission spectrum of the donor and the absorption spectrum of the acceptor, which is calculated by the following Eq (7):

$$J=\frac{\Sigma F\left(\lambda \right)\varepsilon \left(\lambda \right)\lambda^{4}\Delta \lambda }{\Sigma F\left(\lambda \right)\Delta \lambda }$$ (7)

where *F(λ)* is the fluorescence intensity of the donor at wavelength *λ*; *ε(λ)* is the molar absorbance coefficient of the acceptor when the wavelength is *λ*. The overlap of the fluorescence emission spectrum of BSA with the absorption spectrum of SAB/RA was shown in Figure B in S1 File. In present BSA–drug systems, *K* ^2^ = 2/3, *N* = 1.36, *Φ* = 0.15 [46]. Hence, *J* can be estimated by integrating the overlap (Figure B in S1 File) of the absorption spectrum of SAB/RA with the fluorescence emission spectrum of BSA in the absence or presence of Au NPs. All the results were shown in Table 3. The values for *r* and *R*_o_ were within 8 nm, and 0.5*R*_o_ < *r* < 1.5*R*_o_, indicating that the energy transfer from BSA to SAB/RA with or without Au NPs could happen with high probability, and there was a combination of static quenching and non-radiative energy transfer between BSA and SAB/RA regardless of the presence or absence of Au NPs [31,51].

**Table 3:** The distance parameters of different drug-protein systems at 298 K.

System	*J* (cm^3^ L mol^-1^)	*E*	*R*_o_ (nm)	*r* (nm)
BSA-SAB	0.810×10^-14^	0.265	2.434	2.885
BSA-Au4-SAB	0.809×10^-14^	0.223	2.434	2.997
BSA-Au3-SAB	0.809×10^-14^	0.213	2.434	3.027
BSA-Au2-SAB	0.805×10^-14^	0.245	2.432	2.933
BSA-RA	1.011×10^-14^	0.220	2.526	3.118
BSA-Au4-RA	1.010×10^-14^	0.225	2.525	3.102
BSA-Au3-RA	1.010×10^-14^	0.225	2.525	3.104
BSA-Au2-RA	1.004×10^-14^	0.227	2.523	3.093

### Exploring the Effect of Au NPs on the BSA-Drug Interactions

**Fig 6:**
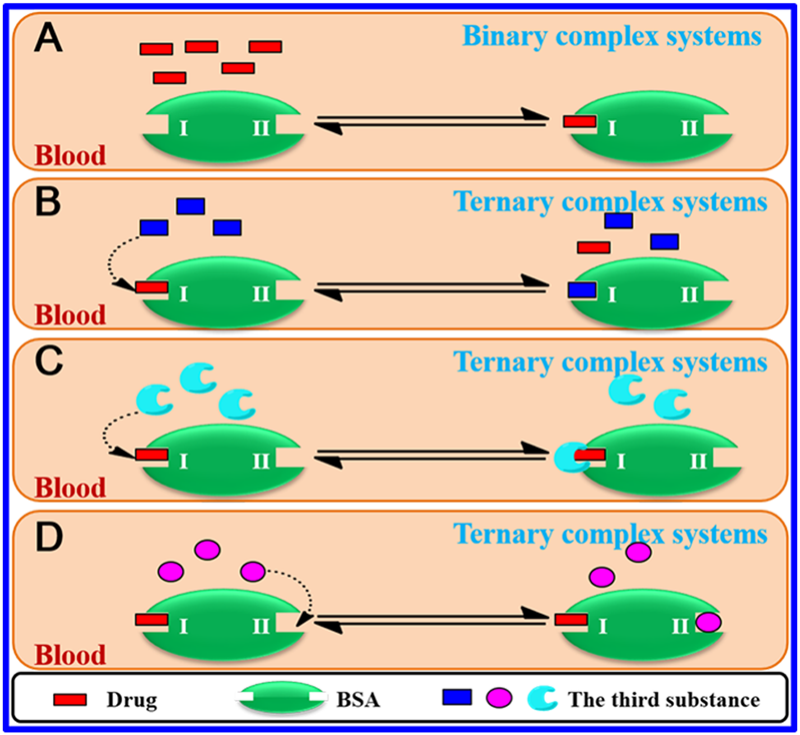
Schematic representation of mechanism of drug binding to BSA in the binary complex systems and the ternary complex systems. (A) The BSA-drug interaction in the binary complex systems. (B) Competitive binding: competitive displacement between the third substance and drug from the same binding site on the BSA in the ternary complex systems. (C) Synergistic reaction: a new complex is formed by the third substance and drug, and then it interacts with BSA in the ternary complex systems. (D) Noncompetitive binding: the interaction of the third substance with BSA at a different binding region induces the conformational change of drug binding site on the BSA in the ternary complex systems.

**Fig 7:**
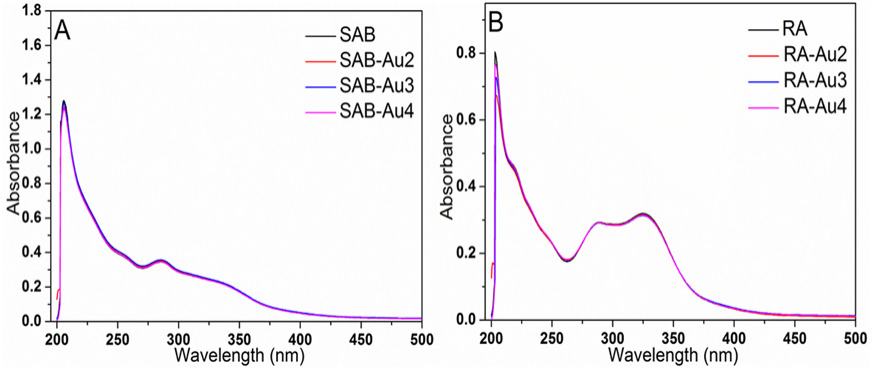
UV-visible absorbance spectra of free drug (A: SAB and B: RA) and their corresponding Au NPs complexes. C_SAB_ = C_RA_ = 2.5 × 10^-5^ mol L^-1^, the concentration of Au NPs was 10.0 × 10^-11^ mol L^-1^.

For the BSA-drug system, addition of the third substance in the above binary system will inevitably break the equilibrium of the original binary system (Fig 6A) and have an impact on their binding mode and binding constant. The possible mechanism for the phenomena can be attributed to three following reasons [52,53]: (1) competitive binding, induced the decrease of the binding affinity between BSA and drug (Fig 6B); (2) synergistic reaction, formed a new complex and influenced the interaction between BSA and drug (Fig 6C); (3) noncompetitive binding, led to the conformational change of BSA, which then changed the binding capability of drug to BSA (Fig 6D). In this experiment, Au NPs were first incubated with the BSA solution for a while, and then the drug was added the above binary system. Hence Au NPs first combined with BSA to form BSA-Au NPs complex, and afterwards the drug reacted with BSA-Au NPs complex. In order to investigate the effect of addition of Au NPs on the binding of drug to BSA, the three reasons mentioned above were carefully studied and analyzed. For the first reason, we discussed the possibility for such phenomena to appear. If there existed the competitive binding between Au NPs and drug, the binding distance between BSA and drug in the presence of Au NPs would have smaller change from that in the absence of Au NPs. However, according to the Table 3, it was obvious that the distance were changed with Au NPs relative to that without Au NPs. So it can be concluded that the first reason may impossible. For the second reason, from Fig 7 it could be seen that the UV-vis absorption spectra of SAB/RA and the difference absorption spectra between SAB/RA-Au NPs and Au NPs at the same concentration could be superposed within experimental error, which verified that the interaction between drug and Au NPs cannot occur, that is, a new drug-Au NPs complex did not form. So the second reason did not also appear. Finally, we analyzed the third reason. In the light of Table 2 and Table 3, it was found that the binding constants (*K*_b_) of BSA-SAB system all reduced to different extent and the binding distance (*r*) between BSA and SAB increased in the presence of Au NPs. It may suggest that the increase of binding distance made the combination more difficult, caused by the conformational change of BSA, and the conformational change of BSA was induced by the initial complex formed by Au NPs and BSA. Hence, the Au NPs hindered the interaction between BSA and SAB, and led to the decrease of binding constant. In the same way, the first formation of Au NPs-BSA complex changed the conformation of BSA, which then affected RA binding with BSA. In summary, it was reasonable to assume that Au NPs had noncompetitive binding site with SAB/RA rather than the competitive binding site, which induced the conformational change of BSA and further affected the binding constant and binding mode of drug with BSA.

### Identification of the Binding Sites of SAB/RA on BSA

**Fig 8:**
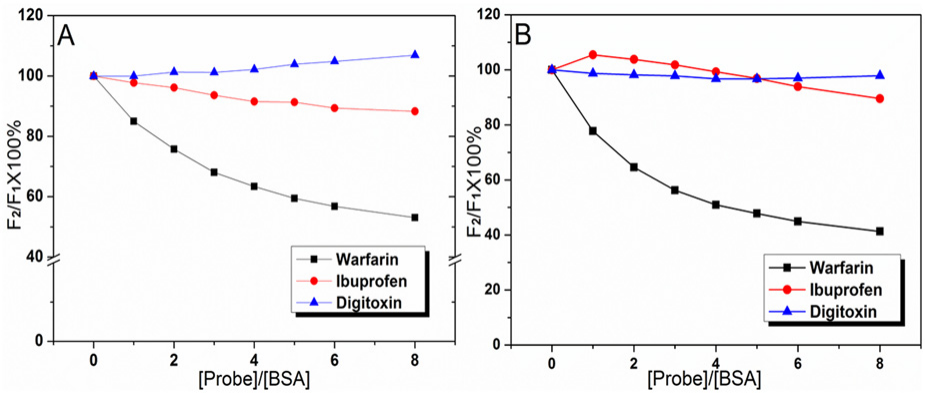
Effect of site marker probes on the fluorescence intensities of BSA-drug systems (A: BSA+SAB, B: BSA+RA). Site marker competitive experiments were carried out using three site markers (warfarin, ibuprofen, and digitoxin). C_BSA_ = 5.0 × 10^-6^ mol L^-1^, C_SAB_ = C_RA_ = 2.0 × 10^-5^ mol L^-1^, *λ*_ex_ = 280 nm, T = 298 K.

BSA is considered to be a heart-shaped helical monomer composed of three structurally homologous *α*-helices domains (I, II, III), and each domain is composed of two subdomains (A and B) [25]. In order to substantiate drug binding site on the BSA, competitive binding between the drug and other ligands that specifically bind to a known site or domain was scrutinized. The exceptional capability of BSA to bind various substances is predominantly dependent on the existence of two primary binding regions, namely Sudlow’s site I and site II, which are located in specialized pockets in subdomain IIA and IIIA, respectively [31,39]. Sudlow et al. [54] have proposed that site I of serum albumin showed affinity for warfarin, phenylbutazone, azapropazone, etc., and site II for ibuprofen, flufenamic acid, diazepam, etc. The binding of digitoxin was found to be independent of sites I and II, which was defined as site III [39]. In this paper, the primary binding site of SAB/RA to BSA was confirmed at 298 K by competitive experiments using warfarin, ibuprofen, and digitoxin as site markers. Firstly, site markers were gradually added to the mixture solution of BSA-drug, and the ratio of drug to BSA was kept at 4:1 to maintain the nonspecific binding of probes to a minimum. Then, the percentage of displacement of the fluorescence probe displaced by the drug was determined according to the method of Sudlow et al. [55]:

$$\text{Probe displacement }\left(\text{\%}\right)=\;\frac{F_{2}}{F_{1}}\;\times \;100\%$$ (8)

where *F*_1_ and *F*_2_ are the fluorescence of BSA-drug in the absence and presence of the probe, respectively. The changes in the fluorescence intensities upon the additions of three site probes were represented in Fig 8. It was clear that the fluorescence intensities of BSA-drug systems decreased markedly after the addition of warfarin, but for other two probes (ibuprofen and digitoxin) the fluorescence intensities had no obvious changes. These results indicated that warfarin displaced SAB/RA from the binding site I. Hence, it could be concluded that SAB and RA were mainly bound to the hydrophobic pocket of Sudlow Site I in subdomain IIA of BSA, namely Trp-212 was near or within the binding site of SAB/RA, which was in accordance with the previous result [55].

### Synchronous Fluorescence Spectroscopy

It was ascertained that the binding of drug to BSA caused the fluorescence quenching, but it was a still a puzzle whether this binding affected the microenvironment near the fluorophore of BSA. So the method of synchronous fluorescence was used. Synchronous fluorescence spectroscopy can provide information about the molecular microenvironment in the vicinity of the fluorophore of molecules [56]. The shift in maximum emission wavelength reflects the change of polarity around chromophore of molecule [46]. The excitation and emission wavelength interval (Δ*λ*) were fixed at 15 and 60 nm, at which the spectrum showed the particular information of tyrosine and tryptophan residues in BSA, respectively [56].

**Fig 9:**
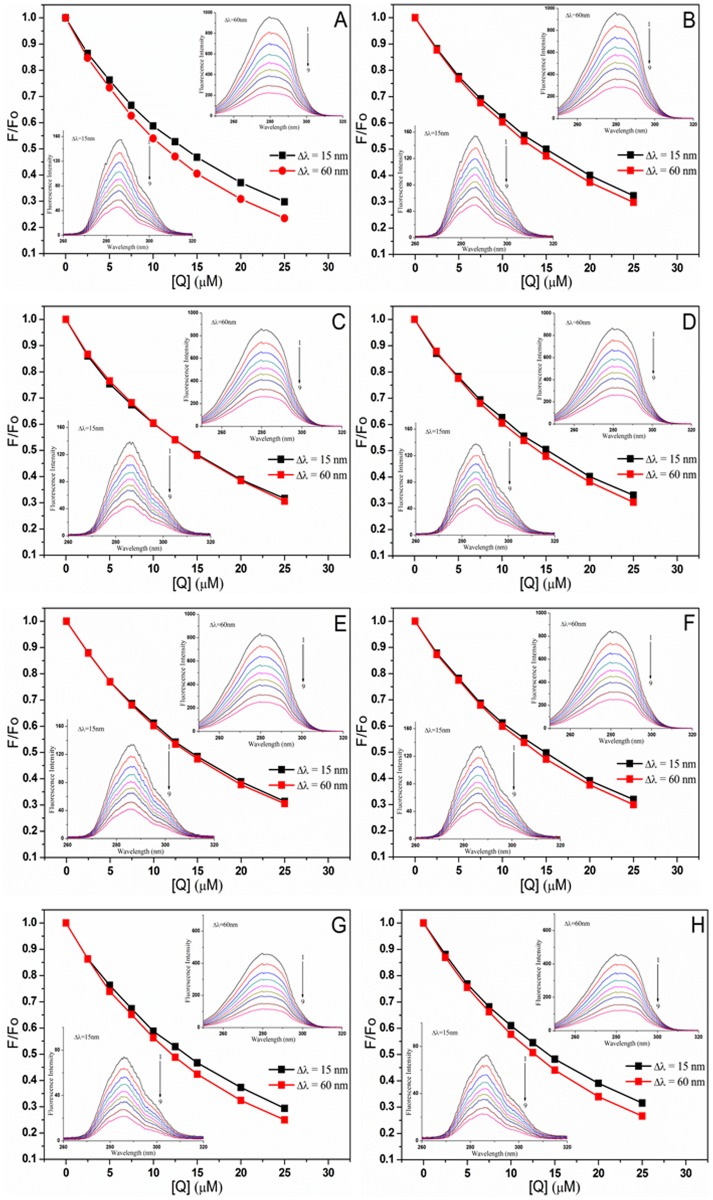
The synchronous fluorescence spectra of BSA–drug systems without and with Au NPs at different Δ*λ* values (15 and 60 nm). (A) BSA-SAB system, (B) BSA-RA system, (C) BSA-SAB-Au4 system, (D) BSA-RA-Au4 system, (E) BSA-SAB-Au3 system, (F) BSA-RA-Au3 system, (G) BSA-SAB-Au2 system and (H) BSA-RA-Au2 system. The concentration of BSA was fixed at 5.0 × 10^-6^ mol L^-1^ and the concentrations of SAB/RA (1–9) were 0, 2.5, 5.0, 7.5, 10.0, 12.5, 15.0, 20.0 and 25.0 × 10^-6^ mol L^-1^, respectively. The concentration of Au NPs was 10.0 × 10^-11^ mol L^-1^.

The effects of SAB/RA on the synchronous fluorescence spectra of BSA with or without Au NPs were shown in Fig 9. It was apparent that an increase in SAB/RA concentration from 0 to 25.0 × 10^-6^ mol L^-1^ could lead to the decrease of fluorescence intensity regularly in all systems, which further demonstrated the occurrence of fluorescence quenching and the interaction between BSA and drug. Moreover, the emission maximum of tyrosine and tryptophan residues showed almost no shift at the investigated concentration, indicating that the polarity around fluorophore in BSA had no obvious change during the binding process. A similar fluorescence quenching profiles were found for both tyrosine and tryptophan residues upon addition of SAB/RA to BSA in the presence and absence of Au NPs, which could be explained that tyrosine and tryptophan residues of BSA might have equal accessibility to SAB/RA. A tyrosine residue such as Tyr-263 is located in domain II of BSA with a hydrophobic environment, and Trp-212 is also located in this domain [57]. The fluorescence changes suggested that the interaction between drug and BSA may occur in domain II [58].

### Three-Dimensional Fluorescence Spectroscopy

The three-dimensional fluorescence spectroscopy is a powerful method to study the conformational change of protein in recent years. If there are changes at the excitation or emission wavelength of the fluorescence peaks, i.e. emergence of a new peak, disappearance of some present peaks and so forth, it can speculate that the conformation of protein has undergone significant changes during the binding process [31,56,58]. The three-dimensional fluorescence spectra and contour maps of BSA and BSA-drug systems in the absence and presence of Au NPs were shown in Figure C in S1 File and the corresponding characteristic parameters were listed in Table 4. As can be seen from Figure C in S1 File, all the three-dimensional fluorescence spectra had four peaks. Peak a (*λ*_em_ = *λ*_ex_) was the Rayleigh scattering peak, and Peak b (*λ*_em_ = 2*λ*_ex_) represented the second-order scattering peak. In addition to two scattering peaks, another two typical fluorescence peaks 1 and 2 were found in the middle of the figure. Peak 1 (*λ*_ex_ = 280.0 nm, *λ*_em_ = 348 nm) mainly displayed the spectral behavior of tryptophan and tyrosine residues, because at 280 nm the fluorescence spectra of protein were known to be characterized by intrinsic fluorescence of tryptophan and tyrosine residues, and the fluorescence of phenylalanine residue being negligible [46,56]. On the other hand, Peak 2 (*λ*_ex_ = 230.0 nm, *λ*_em_ = 347 nm) was related to the fluorescence character of polypeptide backbone structure of BSA aroused by the *P*→P* transition of structure C = O in protein, and reflected changes in the secondary structure of BSA [26]. In the light of this, any changes in Peak 1 and Peak 2 showed the tertiarily and secondarily structural alterations, respectively [56]. The fluorescence intensities of both Peak 1 and Peak 2 after the addition of SAB/RA in spite of the presence or absence of Au NPs decreased to different extents along with a red shift, which meant that the structure of BSA was changed, that is, the microenvironment and conformation had undergone significant alterations during the formation of BSA-drug complex. Moreover, compared BSA-Au NPs systems with the single BSA system, it can be found that the Au NPs resulted in a conformational change of BSA, and then influenced binding activity between BSA and SAB/RA. Table 4 also showed that the intensities and positions (*λ*_ex_ and *λ*_em_) of Peak 1 and Peak 2 were changed in a different way for three different sizes of Au NPs, indicating that each kind of Au NPs had its own special effect on the BSA-drug interaction. In a word, from the above analysis it can be concluded that the interaction between SAB/RA and BSA caused the conformational and microenvironmental alterations of BSA.

**Table 4:** Three-dimensional fluorescence spectral characteristics of BSA-drug systems.

Systems	Peaks	Peak position	Stokes	Intensity
		*λ*_ex_/*λ*_em_ (nm/nm)	Δ*λ* (nm)	*F*
BSA	peak a	240/240→360/360	0	51.2→269.8
	peak 1	280.0/348.0	68	995.8
	peak 2	230.0/347.0	117	398.0
BSA-SAB	peak a	240/240→360/360	0	29.6→261.2
	peak 1	280.0/349.0	69	526.4
	peak 2	230.0/351.0	121	207.5
BSA-RA	peak a	240/240→360/360	0	33.7→250.8
	peak 1	280.0/352.0	72	602.7
	peak 2	230.0/350.5	120.5	253.0
BSA-Au4	peak a	240/240→360/360	0	73.6→406.8
	peak 1	280.0/347.5	67.5	858.7
	peak 2	230.0/344.0	114	361.1
BSA-Au4-SAB	peak a	240/240→360/360	0	44.0→397.7
	peak 1	280.0/350.5	70.5	529.8
	peak 2	230.0/351.0	121	207.8
BSA-Au4-RA	peak a	240/240→360/360	0	49.0→382.6
	peak 1	280.0/351.0	71	544.5
	peak 2	230.0/349.0	119	226.1
BSA-Au3	peak a	240/240→360/360	0	97.2→651.5
	peak 1	280.0/348.5	68.5	835.0
	peak 2	230.0/345.0	115	352.0
BSA-Au3-SAB	peak a	240/240→360/360	0	54.7→573.9
	peak 1	280.0/349.5	69.5	514.1
	peak 2	230.0/349.5	119.5	203.9
BSA-Au3-RA	peak a	240/240→360/360	0	60.4→571.4
	peak 1	280.0/351.0	71	529.7
	peak 2	230.0/350.0	120	220.3
BSA-Au2	peak a	240/240→360/360	0	252.5→977.6
	peak 1	280.0/349.0	69.0	464.2
	peak 2	230.0/344.0	114	208.5
BSA-Au2-SAB	peak a	240/240→360/360	0	152.9→977.6
	peak 1	280.0/349.5	69.5	268.2
	peak 2	230.0/347.5	117.5	114.2
BSA-Au2-RA	peak a	240/240→360/360	0	176.4→982.4
	peak 1	280.0/350.0	70	276.8
	peak 2	230.0/351.5	121.5	125.2

## Conclusions

In this paper, the binding mechanisms of SAB/RA interacting with BSA in the presence and absence of Au NPs with different diameters were investigated by spectroscopic methods. Analysis of fluorescence quenching data of BSA by the drugs revealed that both dynamic and static quenching were involved for the binding process, but the static quenching mechanism was the dominant one. The binding affinity of SAB to BSA with Au NPs was lower than that without Au NPs. Meanwhile, with decreasing the size of Au NPs, the binding affinity was reduced. However, for BSA-RA system the binding affinity was enhanced with increasing Au NPs size. The conformational change of BSA caused by the addition of Au NPs may be the main reason for the changed binding affinity of drugs for BSA. The stoichiometry of binding (n) equals to 1, so the molar ratio of BSA-drug is 1:1 irrespective of the absence or presence of Au NPs, when they are combined into a new complex. Thermodynamic parameters indicated that binding was a spontaneous process. The fluorescence competitive experiments suggested that SAB and RA were mainly bound to subdomain IIA (site I) of BSA. The interactions between SAB/RA and BSA with or without Au NPs will induce structural changes in the conformation and microenvironment of BSA. All of these results will be beneficial for investigating the pharmacokinetic and pharmacodynamics behavior of SAB/RA in pharmacology and clinical medicine.

## Supporting Information

S1 File **Figure A, Fluorescence titration curves of BSA in the presence of SAB/RA at λ_ex_ = 280 and 295 nm.** C_BSA_ = 5.0 × 10^-6^ mol L^-1^, T = 298 K, pH = 7.4. (A): BSA-SAB system, (B): BSA-RA system, (C): BSA-SAB-Au4 system, (D): BSA-RA-Au4 system, (E): BSA-SAB-Au3 system, (F): BSA-RA-Au3 system, (G): BSA-SAB-Au2 system, (H): BSA-RA-Au2 system. **Figure B, Overlapping of the fluorescence emission spectrum of BSA (1) with the absorption spectrum of drug (2).** (A): BSA-SAB system, (B): BSA-RA system, (C): BSA-SAB-Au4 system, (D): BSA-RA-Au4 system, (E): BSA-SAB-Au3 system, (F): BSA-RA-Au3 system, (G): BSA-SAB-Au2 system, (H): BSA-RA-Au2 system. The concentrations of drug and BSA were 5.0 × 10^-6^ mol L^-1^ and the concentration of Au NPs were 10.0 × 10^-11^ mol L^-1^, *λ*_ex_ = 280 nm, T = 298 K. **Figure C, The three-dimensional fluorescence spectra (A, B, C, D, E, F, G, H, I, J, K, L) and corresponding contour spectra (A’, B’, C’, D’, E’, F’, G’, H’, I’, J’, K’, L’) of drug-BSA systems.** (A, A’): BSA system, (B, B’): BSA-SAB system, (C, C’): BSA-RA system, (D, D’): BSA-Au4 system, (E, E’): BSA-SAB-Au4 system, (F, F’): BSA-RA-Au4 system, (G, G’): BSA-Au3 system, (H, H’): BSA-SAB-Au3 system, (I, I’): BSA-RA-Au3 system, (J, J’): BSA-Au2 system, (K, K’): BSA-SAB-Au2 system, (L, L’): BSA-RA-Au2 system. C_BSA_ = 5.0 × 10^-6^ mol L^-1^, C_SAB_ = C_RA_ = 10.0 × 10^-6^ mol L^-1^, and the concentration of Au NPs were 10.0 × 10^-11^ mol L^-1^.
(DOCX)
