# Supplementary material for: Elucidating the Influence of Gold Nanoparticles on the Binding of Salvianolic Acid B and Rosmarinic Acid to Bovine Serum Albumin
Source: PLoS One. 2015 Apr 10;10(4):e0118274. doi: 10.1371/journal.pone.0118274 (PMC4393081; doi:10.1371/journal.pone.0118274)
Supplement: S1 File — Figure A, Fluorescence titration curves of BSA in the presence of SAB/RA at λex = 280 and 295 nm. CBSA = 5.0 × 10^-6^ mol L-1, T = 298 K, pH = 7.4. (A): BSA-SAB system, (B): BSA-RA system, (C): BSA-SAB-Au4 system, (D): BSA-RA-Au4 system, (E): BSA-SAB-Au3 system, (F): BSA-RA-Au3 system, (G): BSA-SAB-Au2 system, (H): BSA-RA-Au2 system. Figure B, Overlapping of the fluorescence emission spectrum of BSA (1) with the absorption spectrum of drug (2). (A): BSA-SAB system, (B): BSA-RA system, (C): BSA-SAB-Au4 system, (D): BSA-RA-Au4 system, (E): BSA-SAB-Au3 system, (F): BSA-RA-Au3 system, (G): BSA-SAB-Au2 system, (H): BSA-RA-Au2 system. The concentrations of drug and BSA were 5.0 × 10^-6^ mol L-1 and the concentration of Au NPs were 10.0 × 10-11 mol L-1, λ ex = 280 nm, T = 298 K. Figure C, The three-dimensional fluorescence spectra (A, B, C, D, E, F, G, H, I, J, K, L) and corresponding contour spectra (A’, B’, C’, D’, E’, F’, G’, H’, I’, J’, K’, L’) of drug-BSA systems. (A, A’): BSA system, (B, B’): BSA-SAB system, (C, C’): BSA-RA system, (D, D’): BSA-Au4 system, (E, E’): BSA-SAB-Au4 system, (F, F’): BSA-RA-Au4 system, (G, G’): BSA-Au3 system, (H, H’): BSA-SAB-Au3 system, (I, I’): BSA-RA-Au3 system, (J, J’): BSA-Au2 system, (K, K’): BSA-SAB-Au2 system, (L, L’): BSA-RA-Au2 system. CBSA = 5.0 × 10^-6^ mol L-1, CSAB = CRA = 10.0 × 10-6 mol L-1, and the concentration of Au NPs were 10.0 × 10-11 mol L-1. (DOCX) [file pone.0118274.s001.docx]

***Supporting Information***

**Elucidating the influence of gold nanoparticles on the binding of salvianolic acid B and rosmarinic acid to bovine serum albumin**

**Xin Peng^a,b^  Wei Qi^b,c,d,e^* Renliang Huang^f^ Rongxin Su^b,c,d,e^ Zhimin He^b,c^**

^a^ *School of Life Sciences, Tianjin University, Tianjin 300072, PR China*

^b^ *Chemical Engineering Research Center, School of Chemical Engineering and Technology, Tianjin University, Tianjin 300072, PR China*

^c^ *State Key Laboratory of Chemical Engineering, Tianjin University, Tianjin 300072, PR China*

^d^ *Tianjin Key Laboratory of Membrane Science and Desalination Technology, Tianjin University, Tianjin 300072, PR China*

^e^ *Collaborative Innovation Center of Chemical Science and Engineering (Tianjin), Tianjin 300072, P. R. China*

*^f^ School of Environmental Science and Engineering, Tianjin University, Tianjin 300072, PR China*

_________________________________________________

*Corresponding author: Wei Qi

Address: Chemical Engineering Research Center, School of Chemical Engineering and Technology, Tianjin University, Tianjin 300072, PR China

Tel: +86-22-27407799

Fax: +86-22-27407599

E-mail: qiwei@tju.edu.cn (W. Qi)






















**Figure A. Fluorescence titration curves of BSA in the presence of SAB/RA at λ_ex_ = 280 and 295 nm.** C_BSA_ **=** 5.0 × 10^–6^ mol L^-1^, T = 298 K, pH = 7.4. (A): BSA-SAB system, (B): BSA-RA system, (C): BSA-SAB-Au4 system, (D): BSA-RA-Au4 system, (E): BSA-SAB-Au3 system, (F): BSA-RA-Au3 system, (G): BSA-SAB-Au2 system, (H): BSA-RA-Au2 system.












**



**







**Figure B.**  **Overlapping of the fluorescence emission spectrum of BSA (1) with the absorption spectrum of drug (2).** (A): BSA-SAB system, (B): BSA-RA system, (C): BSA-SAB-Au4 system, (D): BSA-RA-Au4 system, (E): BSA-SAB-Au3 system, (F): BSA-RA-Au3 system, (G): BSA-SAB-Au2 system, (H): BSA-RA-Au2 system. The concentrations of drug and BSA were 5.0 × 10^–6^ mol L^-1^ and the concentration of Au NPs were 10.0 × 10^–11^ mol L^-1^, *λ*_ex_ = 280 nm, T = 298 K.






























































**Figure C. The three-dimensional fluorescence spectra (A, B, C, D, E, F, G, H, I, J, K, L) and corresponding contour spectra (A’, B’, C’, D’, E’, F’, G’, H’, I’, J’, K’, L’) of drug-BSA systems.** (A, A’): BSA system, (B, B’): BSA-SAB system, (C, C’): BSA-RA system, (D, D’): BSA-Au4 system, (E, E’): BSA-SAB-Au4 system, (F, F’): BSA-RA-Au4 system, (G, G’): BSA-Au3 system, (H, H’): BSA-SAB-Au3 system, (I, I’): BSA-RA-Au3 system, (J, J’): BSA-Au2 system, (K, K’): BSA-SAB-Au2 system, (L, L’): BSA-RA-Au2 system. C_BSA_ = 5.0 × 10^-6^ mol L^-1^, C_SAB_ = C_RA_ = 10.0 × 10^-6^ mol L^-1^, and the concentration of Au NPs were 10.0 × 10^–11^ mol L^-1^.
